# Dynamic succession of the quantity and composition of epiphytic microorganisms at different growth stages on rice surface

**DOI:** 10.3389/fmicb.2024.1451935

**Published:** 2024-11-07

**Authors:** Lijuan Huang, Zhifei Zhang, Lin Mu, Xiong Liu, Rongji Sun, Wenjing Gao, Guihua Chen

**Affiliations:** ^1^College of Agronomy, Hunan Agricultural University, Changsha, China; ^2^Hunan Provincial Key Laboratory of Rice and Rapeseed Breeding for Disease Resistance, Changsha, China

**Keywords:** rice, growth stage, epiphytic microorganisms, quantity, composition, microbial diversity

## Abstract

The quality of silage is uneven, which is due to the difference of epiphytic microorganisms of raw materials. To improve the quality of fermentation, the quantity and composition of epiphytic microorganisms are usually analyzed to better prepare silage. In this research, plate coating method and 16S high-throughput sequencing were used to analyze the differences in the quantity and composition of rice epiphytic microorganisms during different growth stages. The Lactic acid bacteria (LAB) and aerobic bacteria were the highest at the flowering stage, the yeast was the highest at the milk ripening stage, and the mould was the highest at the full ripening stage. And the growth stage also had a great influence on the composition of epiphytic bacterial community, at the phylum level, it was mainly composed of Proteobacteria. And at the genus level, *Pantoea, Acinetobacter, Pseudomonas* and *Chryseobacterium* were dominanted at the flowering stage; *Pantoea, Stenotrophomonas* and *Sphingobacterium* were dominanted at the milk ripening stage; *Acinetobacter, Pantoea, Chryseobacterium* and *Lactococcus* were dominanted at the dough stage; *Acinetobacter* and *Klebsiella* were dominated at the full ripening stage. Overall, the growth stage significantly affected the quantity and composition of rice epiphytic microorganisms. Therefore, rice silage can be modulated reasonably according to the number and composition of epiphytic microorganisms in different growth stages.

## 1 Introduction

Rice (*Oryza sativa* L.), as one of the three major food crops in China, accounts for ~19% of the world's planted area. According to the rice yield and rice straw coefficient of 1.0, the annual rice straw yield in 2022 is approximately 20 million tons (Wang et al., 2010). Most of it was used as fertilizer and living fuel, and a certain amount was also burned and returned to the field, while only 16.2% was used as feed (OuYang et al., 2010). This not only causes a waste of resources but also causes serious pollution to the environment. Recently, the demand for roughage increased greatly with the rise of ensiling, biodegradation, and other ways that can improve rice straw's physical and chemical properties and nutritional quality and promote animal digestion and utilization rate, which not only ensure the supply of animal feed but also increase the economic benefits of enterprises (Cui et al., 2021; Liu et al., 2023). At present, rice silage is one of the important sources of roughage in China. The fermentation process of forage silage is affected by diverse factors, among which epiphytic microorganisms are one of the important factors affecting the fermentation quality of forage silage. With the gradual maturity of forage, the quantity and composition of epiphytic microorganisms change, and these changes will directly affect the quality of forage silage (Tohno et al., 2012). Therefore, it is very important to know the quantity and composition of epiphytic microorganisms in forage grass, which can provide a scientific basis for regulating the fermentation process of silage and preparing high-quality silage (Hou et al., 2023).

The forage grass surface was mainly composed of aerobic bacteria, yeast, mold, and LAB (Lin et al., 1992a). The epiphytic LAB and mold on the rice surface were 1.16 and 4.43 lg10 CFU/g FM, respectively (Wang et al., 2014). The LAB, yeast, mold, and aerobic bacteria on sweet sorghum (*Sorghum bicolor*) surface were 8.59, 7.86, 6.00, and 8.57 lg10 CFU/g FM, respectively (Zhao et al., 2023). The epiphytic LAB on *Phalaris arundinacea* surface were 1.4–1.8 lg10 CFU/g FM; aerobic bacteria, yeast, and mold were 4.6–5.9, 5.2–5.7, and 3.8–4.8 lg10 CFU/g FM, respectively (Zhang et al., 2019). It has been found that there were more undesirable microorganisms and less LAB on the surface of forage grass. Ali et al. (2020) found that the surface of sweet sorghum was dominated by Proteobacteria (96.5%), Firmicutes (2.43%), and Actinomycetes (0.70%) at the phylum level, and *Enterobacter, Rosenbergiella*, and *Erwinia* were dominated at the genus level. Yuan et al. (2020) found that the surface of Napier grass (*Pennisetum purpureum*) was dominated by Proteobacteria (64.88%), Cyanobacteria (16.94%), Firmicutes (6.64%), and Actinomycetes (6.29%) at the phylum level, and the *Enterococcus, Enterobacter*, and *Pediococcus* were dominated at the genus level. Nazar et al. (2021) found that the dominant bacterium genus of whole corn (*Zea mays* L.) was *Enterobacter* (32.8%), while LAB were *Lactococcus* (4.42%), *Weissella* (1.73%), *Leuconostoc* (1.29%), and *Lactobacillus* (0.02%). Wang et al. (2020) found that the dominant epiphytic bacteria of corn at the phylum level were Proteobacteria (86.34%), while the dominant genus was *Enterobacter* (>14%). In general, the low quantity of epiphytic LAB and the high quantity of undesirable microorganisms on the surface of forage grass were not conducive to silage fermentation.

Microbial diversity was one of the key factors affecting the fermentation process of silage (Lei et al., 2023; Jaipolsaen et al., 2022), which provides a reference for the rational utilization of rice silage. However, little was known about the epiphytic microorganism diversity of rice before silage. Xu et al. (2010) detected the microorganisms on the leaf surface of corn at different growth stages and found that the quantity and composition of microorganisms on the leaf surface were different during the whole growth stage, while aerobic bacteria and fungus still dominated. The quantity of aerobic bacteria was the highest at the spinning stage, and the quantity of fungus was the highest at the 4-leaf and 1-heart stages. Lin et al. (1992b) found that the quantity of LAB on alfalfa (*Medicago sativa* L.) surface increased with the progress of the growth stage, and the composition of LAB was significantly different at different growth stages. Many research studies on the diversity of herbage microorganisms have focused on a certain growth stage (Johnson et al., 1999). However, there has been less attention paid to the changes in the quantity and composition of epiphytic microorganisms during the whole growth stage. Therefore, this research hypothesized that the diversity of epiphytic microorganisms at different growth stages of the rice surface may affect the rational utilization of rice at different growth stages. Five different varieties of rice were used as experimental materials, and the samples were taken at the seedling stage, tillering stage, jointing stage, booting stage, heading stage, flowering stage, milk ripening stage, dough stage, and full ripening stage. The quantity of epiphytic LAB, yeast, mold, and aerobic bacteria was determined, and the alpha diversity, beta diversity, and composition of epiphytic microorganisms in different growth stages were analyzed by high-throughput sequencing. The aim of this research was to analyze the effect of the growth stage on the quantity and composition of rice epiphytic microorganisms.

## 2 Materials and methods

### 2.1 Experimental materials and planting site

Five rice varieties, namely, Zhuo 201s/6W1622, Zhuo 201s/6W1003, Zhan 998s/X5H008, Zhan 998s/4W0802, and Zhan 998s/R302, were used as experimental materials, which were provided by the College of Agrology, Hunan Agricultural University. The experimental materials were planted in Yunyuan Base of Hunan Agricultural University (28°11 ′2 ″N, 113°4′ 34″ E) on 3 June 2021, during which conventional water and fertilizer management was adopted. During the planting period (3 June to 8 October 2021), the average temperature is 26.8°C; the highest temperature is in July, with an average temperature of 30.5°C; and the lowest temperature is in October, with an average temperature of 18.5°C.

### 2.2 Sample collection and processing

Five rice varieties were sampled at the seedling stage (SS), tillering stage (TS), jointing stage (JS), booting stage (BS), heading stage (HS), flowering stage (FS), milk ripening stage (MS), dough stage (DS), and full ripening stage (FRS). The specific sampling information is shown in Table 1.

**Table 1:** Specific sampling information.

**Growth stage**	**Sampling time**	**Temperature**
Seedling stage (SS)	28 June 2021	28°C
Tillering stage (TS)	9 July 2021	31°C
Jointing stage (JS)	29 July 2021	30°C
Booting stage (BS)	15 August 2021	26°C
Heading stage (HS)	28 August 2021	28°C
Flowering stage (FS)	5 September 2021	30°C
Milk ripening stage (MS)	15 September 2021	29°C
Dough stage (DS)	30 September 2021	26°C
Full ripening stage (FRS)	8 October 2021	19°C

### 2.3 Sampling and culture-based microbial analyses

The detection of culturable epiphytic microorganisms was performed using the methodology described by Xie et al. (2021). In brief, the sample was divided into two parts: One part was the stem and leaf (SL), and the other part was the ear of rice (E), according to the growth site, and the quantity of epiphytic microorganisms in different growth stages and different growth sites of rice was determined. LAB, yeast, mold, and aerobic bacteria were counted by DeMan, Rogosa and Sharpe (MRS), Rose Bengal Agar, and nutrient agar medium. The quantity of epiphytic microorganisms was expressed as lg10 CFU/g FM (fresh matter, FM), repeated three times.

### 2.4 Microbial community diversity analysis

The samples were processed according to the method provided by Shanghai Peisenol Biotechnology Company. In brief, 10 g of rice samples was shaken with sterile PBS for 30 min; then, each mixture was filtered through two layers of sterile gauze and then filtered with a 0.22-μm organic filter membrane. Each sample was repeated three times, and the collected membrane was frozen at−80°C until DNA was extracted. DNA extraction, PCR amplification, and sequencing services were provided by Shanghai Personal Biotechnology Company. The OMEGA DNA Kit (D5625-02) was used to extract total microbial DNA, and 338F (5′-ACTCCTACGGGAGGCAGCA-3′) and 806R (5′-GGACTACHVGGGTWTCTAAT-3′) were designed to amplify the highly variable V3-V4 region of the bacterial 16S rRNA gene. The Illumina NovaSeq platform was used for double-ended sequencing. QIIME2 (2019.4) software was used to analyze the biological information of the microbiome, decode the original sequence data, and remove primers, and then, the DADA2 plug-in was used to filter, denoise, splice, and remove chimeras. The data were analyzed by the online platform Personalbio GensCloud (https://www.genescloud.cn).

### 2.5 Statistical analyses

Data were analyzed by SPSS 25.0. Differences between means values were determined using one-way analysis of variance (ANOVA); data with a *P-value of* < 0.05 were considered significant. Graphing was performed using the platform Personalbio GensCloud and Origin 2021.

## 3 Results

### 3.1 Dynamic changes of epiphytic microorganism quantity in different growth stages

The quantity of epiphytic microorganisms in different growth stages and growth parts of rice is shown in Table 2. For LAB, SL + FS was significantly higher in the stem and leaf part than in other treatments except SL + H S (*P* < 0.05); in the ear part, E + FS was significantly higher than all other treatments (*P* < 0.05), and E + FS was significantly higher than SL+FS (*P* < 0.05). For yeast, SL + MS was significantly higher than other treatments except SL + FRS in the stem and leaf part (*P* < 0.05); in the ear part, E + MS was significantly higher than E + HS and E + FS (*P* < 0.05), while E + MS was higher than SL + MS (*P* < 0.05). For mold, SL + FRS was significantly higher than other treatments except SL + MS in the stem and leaf part (*P* < 0.05), E + FRS was significantly higher than all other treatments in the ear part (*P* < 0.05), and E + FRS was higher than SL + FRS (*P* < 0.05). For aerobic bacteria, in the stem and leaf part, SL + FS was significantly higher than other treatments except SL + MS and SL + FRS (*P* < 0.05); in the ear part, E + FS was significantly higher than other treatments except E + MS (*P* < 0.05), and E + FS was significantly higher than SL + FS (*P* < 0.05).

**Table 2:** Quantity of epiphytic microorganisms in different growth stages and sites (lg10 CFU/g FM).

**Growth site**	**Group**	**LAB**	**Yeast**	**Mold**	**Aerobic bacteria**
SL	SS	2.98 ± 0.16^i^	3.11 ± 0.47^h^	2.16 ± 0.43^g^	5.89 ± 0.24^e^
	TS	3.49 ± 0.34^h^	3.52 ± 0.21^g^	2.78 ± 0.16^f^	6.75 ± 0.35^d^
	JS	4.16 ± 0.22^g^	3.43 ± 0.18^gh^	2.39 ± 0.35^g^	6.96 ± 0.32^cd^
	BS	4.59 ± 0.35^ef^	3.51 ± 0.10^g^	2.97 ± 0.37^f^	6.16 ± 0.22^e^
	HS	4.87 ± 0.14^de^	4.26 ± 0.34^f^	3.47 ± 0.15^e^	6.57 ± 0.54^d^
	FS	5.20 ± 0.13^cd^	4.71 ± 0.31^e^	3.86 ± 0.26^d^	7.39 ± 0.33^bc^
	MS	4.33 ± 0.26^fg^	5.22 ± 0.28^bc^	4.63 ± 0.36^bc^	7.32 ± 0.13^bc^
	DS	4.17 ± 0.30^g^	4.79 ± 0.15^de^	4.52 ± 0.21^c^	6.74 ± 0.23^d^
	FRS	4.09 ± 0.63^g^	5.10 ± 0.24^cd^	5.01 ± 0.21^ab^	6.99 ± 0.29^bcd^
E	HS	4.63 ± 0.51^ef^	4.06 ± 0.33^f^	3.11 ± 0.46^ef^	7.33 ± 0.64^bc^
	FS	6.38 ± 0.21^a^	4.96 ± 0.25^cde^	4.12 ± 0.40^d^	8.65 ± 0.21^a^
	MS	5.94 ± 0.20^b^	5.77 ± 0.07^a^	4.71 ± 0.08^bc^	8.37 ± 0.11^a^
	DS	5.37 ± 0.19^c^	5.60 ± 0.08^a^	4.74 ± 0.19^bc^	7.39 ± 0.18^bc^
	FRS	5.25 ± 0.17^cd^	5.46 ± 0.23^ab^	5.26 ± 0.25^a^	7.45 ± 0.28^b^

SS, seedling stage; TS, tillering stage; JS, jointing stage; BS, booting stage; HS, heading stage; FS, flowering stage; MS, milk ripening stage; DS, dough stage; FRS, full ripening stage; SL, stem and leaf; E, ear. All analyses were carried out in 15 replicates. A P < 0.05 was considered a statistically significant difference. Superscript letters demonstrated significant differences at a P < 0.05 within the same column.

### 3.2 Dynamic changes of bacterial community diversity at different growth stages

According to the results of the epiphytic microorganism count, FS, MS, DS, and FRS treatment groups were selected for high-throughput sequencing analysis. The results showed that a total of 7,154,276 original sequences were obtained, with an average of 119,238 sequences per treatment. After quality filtering, noise removal, and chimera removal, a total of 4,364,264 high-quality sequences were obtained, and an average of 72,738 high-quality sequences were obtained per treatment, that is, asv feature sequences.

#### 3.2.1 Alpha diversity analysis

**Figure 1:**
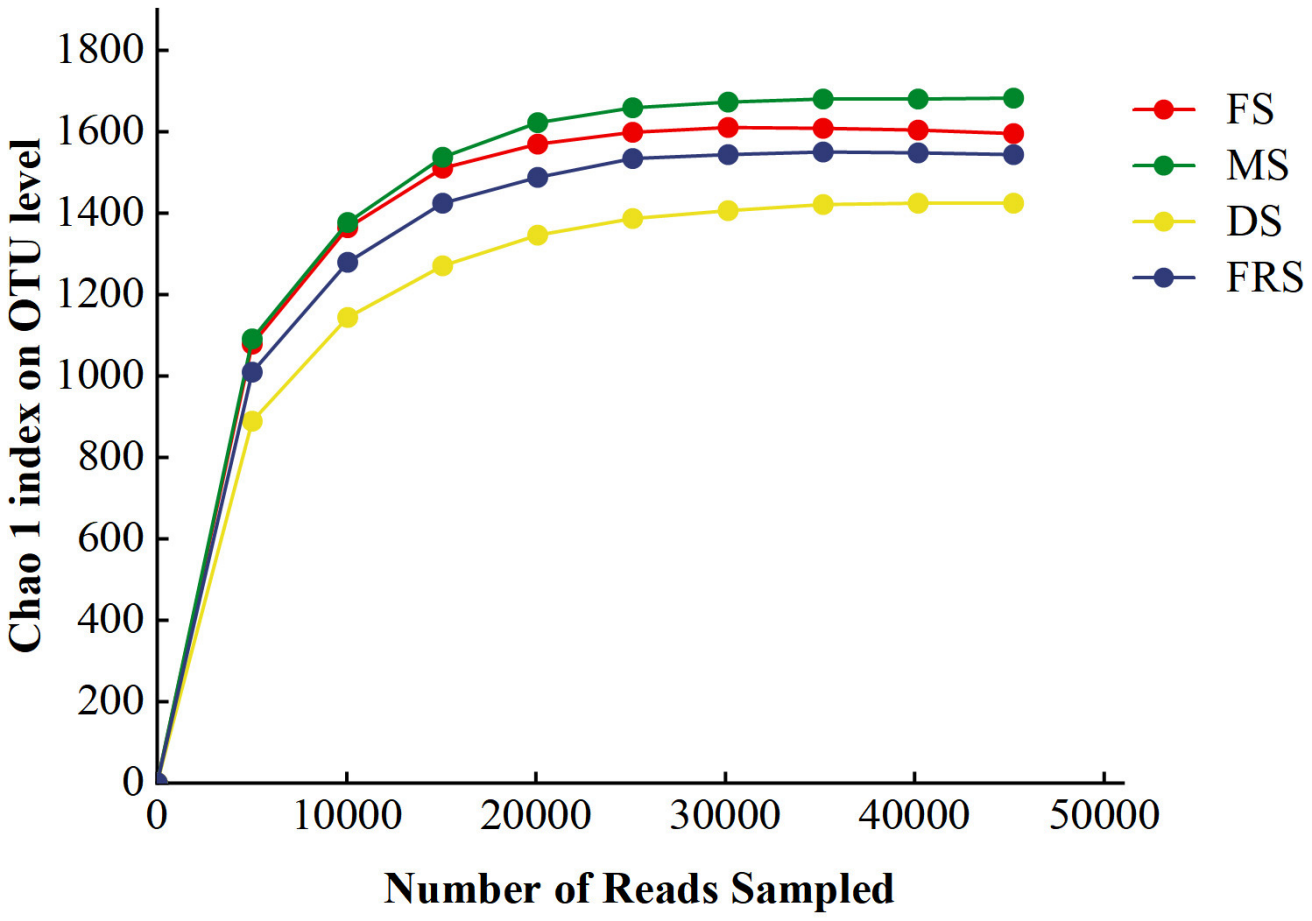
Sample rarefaction curves. FS, flowering stage; MS, milk ripening stage; DS, dough stage; FRS, full ripening stage.

The rarefaction curves of samples are shown in Figure 1, and the Good's coverage index of samples is shown in Table 3. It is obvious that the Good's coverage of all samples is >0.990, and the sparse curve tends to be flat, indicating that the sample sequence is sufficient and the data volume is sufficient to reflect the richness and diversity of species.

**Table 3:** Alpha diversity of rice surface bacterial communities at different growth stages.

**Group**	**Chao1**	**Observed species**	**Shannon**	**Simpson**	**Good's coverage**
FS	1,595.61	1,425.49	6.11	0.91	0.993
MS	1,682.40	1,469.23	6.30	0.93	0.992
DS	1,424.48	1,206.65	5.53	0.88	0.993
FRS	1,544.02	1,348.58	5.89	0.92	0.992

FS, flowering stage; MS, milk ripening stage; DS, dough stage; FRS, full ripening stage. All analyses were carried out in fifteen replicates.

The Chao1 and observed species indices can reveal the richness of bacterial community, while the Shannon and Simpson indices can reveal the diversity of bacterial community. As shown in Table 3, there is no significant difference between the Chao1, observed species, Shannon, and Simpson indices of each processing. The indices of Chao1, observed species, Shannon, and Simpson showed a tendency of first decreasing and then increasing, and they decreased in DS.

#### 3.2.2 Beta diversity analysis

**Figure 2:**
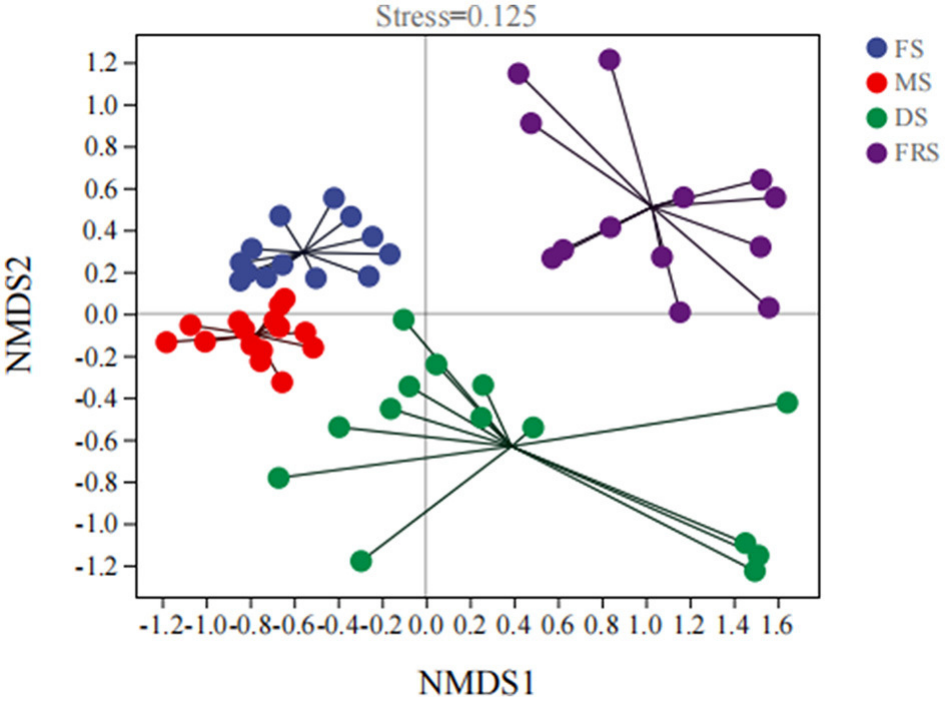
Analysis of beta diversity of epiphytic bacteria at different growth stages of rice. FS, flowering stage; MS, milk ripening stage; DS, dough stage; FRS, full ripening stage.

The analysis of the beta diversity of rice epiphytic microorganisms at different growth stages is shown in Figure 2. Non-metric multidimensional scaling (NMDS) analysis was performed on all samples based on the Bray–Curtis distance algorithm. The degree of difference between different samples is shown in the figure by the distance from point to point. The greater the distance between the two points, the greater the difference in the composition of the microbial communities in the two samples. The stress value of NMDS analysis is 0.125, indicating that the results are reliable and have certain explanatory significance. Among them, the distance between DS and FRS was far, indicating that the epiphytic microorganism composition of the dough stage was different from the full ripening stage, which may be due to the change in abundance and diversity of epiphytic microorganisms.

#### 3.2.3 Analysis of bacterial community composition

**Figure 3:**
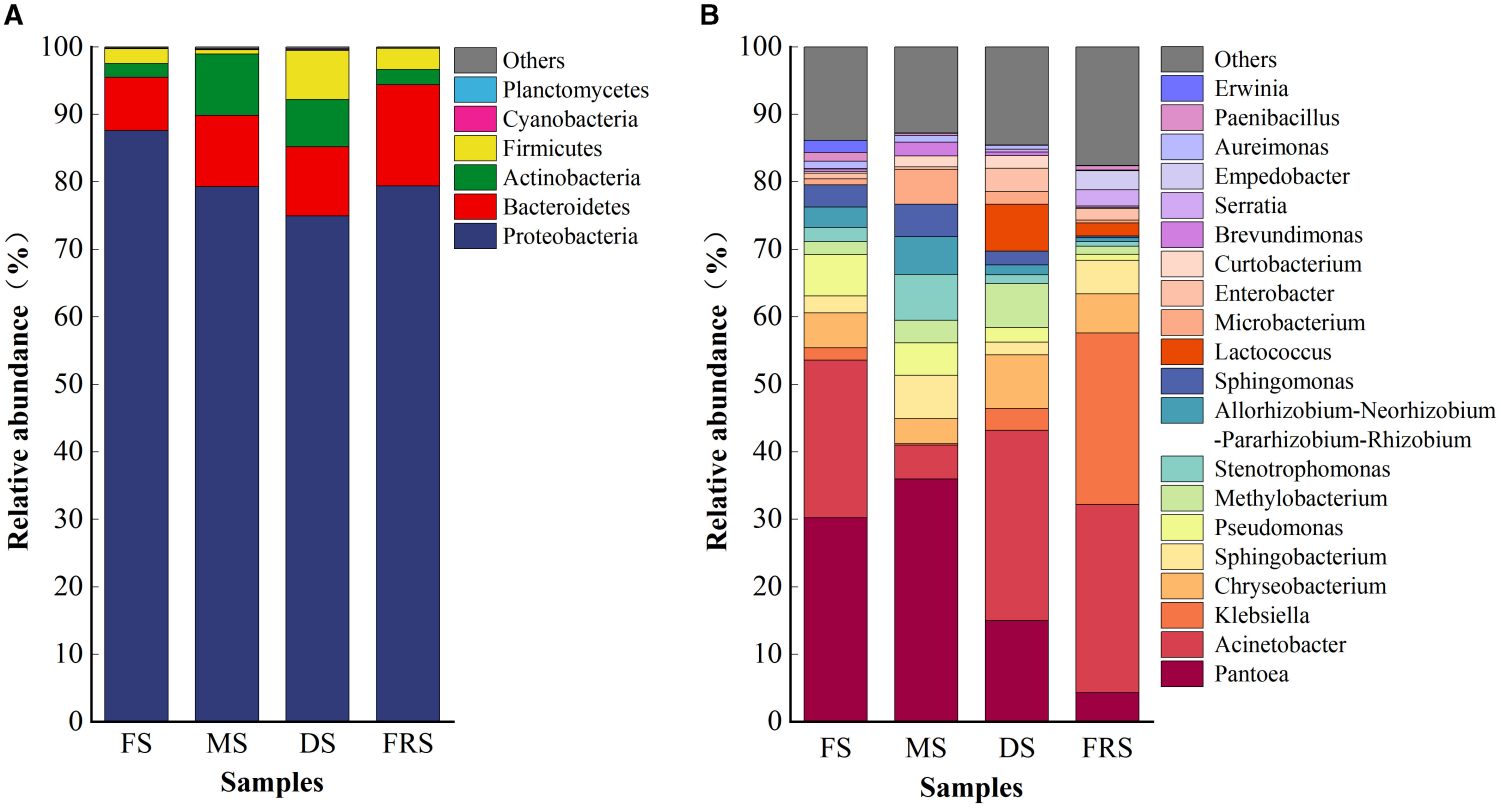
Composition of rice epiphytic bacterial community at different growth stages. **(A)** Relative abundance of bacterial communities at the phylum level. **(B)** Relative abundance of bacterial communities at the genus level (top 20). FS, flowering stage; MS, milk ripening stage; DS, dough stage; FRS, full ripening stage.

The operational taxonomic unit (OTU) sequences obtained from rice samples in each group were annotated from phylum and genus levels to obtain the species classification information of each OTU at different classification levels, and the community structure of the samples at different classification levels was observed. As shown in Figure 3, at the phylum level, the epiphytic bacteria were composed of Proteobacteria, Bacteroidetes, Actinobacteria, and Firmicutes. Proteobacteria dominated in all treatments, with a relative abundance of more than 67%. In FS, the relative abundances of Proteobacteria and Bacteroidetes were 87.58% and 7.92%, respectively. Compared with FS, the relative abundances of Proteobacteria (79.31%) decreased, and the relative abundances of Bacteroides (10.53%) and Actinobacteria (9.11%) increased in MS. Compared with MS, the relative abundances of Proteobacteria, Bacteroidetes, and Actinobacteria decreased in DS, which were 74.97%, 10.24%, and 7.01%. In addition, a higher abundance of Firmicutes (7.25%) was detected in DS compared to other treatments. Compared with DS, the relative abundances of Proteobacteria (79.41%) and Bacteroidetes (14.96%) were higher in FRS, and the relative abundances of Actinobacteria (2.30%) and Firmicutes (3.12%) were lower in FRS.

At the genus level, the dominant bacteria in FS were *Pantoea, Acinetobacter, Pseudomonas*, and *Chryseobacterium*, with relative abundances of 30.23%, 23.34%, 6.06%, and 5.17%, respectively. *Pantoea, Stenotrophomonas, Sphingobacterium, Allorhizobium–Neorhizobium–Pararhizobium– Rhizobium, Micro-bacterium*, and *Acinetobacter* were dominant bacteria in MS, with relative abundances of 35.97%, 6.79%, 6.41%, 5.61%, 5.20%, and 5.00%, respectively. The dominant bacteria in DS were *Acinetobacter, Pantoea, Chryseobacterium, Lactococcus*, and *Methylobacterium*, with relative abundances of 28.15%, 15.01%, 7.93%, 6.94%, and 6.56%, respectively. The dominant bacteria in FRS were *Acinetobacter, Klebsiella*, and *Chryseobacterium*, with relative abundances of 27.92%, 25.40%, and 5.76%, respectively.

#### 3.2.4 Analysis of species differences and marker species

**Figure 4:**
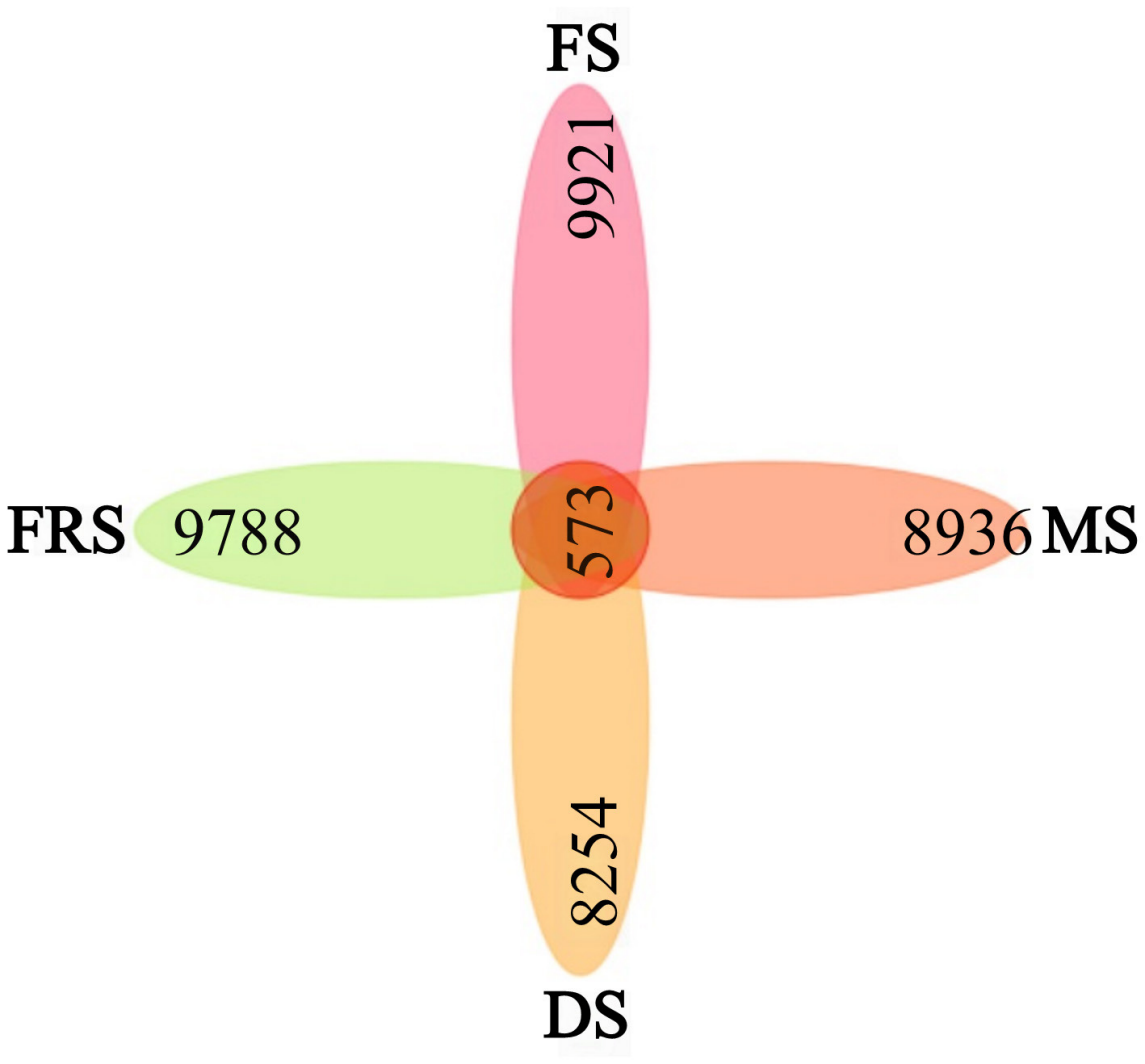
OTU Vane map of rice epiphytic bacteria at different growth stages. Each block represents a group, the overlapping area between the blocks indicates the OTU shared between the corresponding groups, and the number of each block indicates the number of OTU contained in that block. FS, flowering stage; MS, milk ripening stage; DS, dough stage; FRS, full ripening stage.

OTUs are used to classify sequences in rice samples. In general, different 16S rRNA sequences with similarities higher than 97% are defined as one OTU, and each OTU is generally regarded as a microbial species (Zhang et al., 2023). The petal diagram was drawn according to the OTU cluster analysis results. A petal diagram is a way to show the number of unique and common OTUs between samples/groups, each petal represents a group of samples, the core number represents the number of common OTUs in each group, and the number on each petal represents the number of OTUs unique to this group. As shown in Figure 4, the total number of OTUs in rice samples was 573, among which the average number of OTUs in specific groups was as follows: flowering stage (FS): 9,921; milk ripening stage (MS): 8,936; dough stage (DS): 8,254; and full ripening stage (FRS): 9,788. The results of the petal diagram show that, in the tested samples, the OTUs were the most abundant at the flowering stage and gradually decreased at the milk ripening stage and dough stage. When at the full ripening stage, the number of the OTUs increased, more than that at the dough stage and milk ripening stage, indicating that the species diversity on the surface of rice was more abundant than in the early ripening stage.

**Figure 5:**
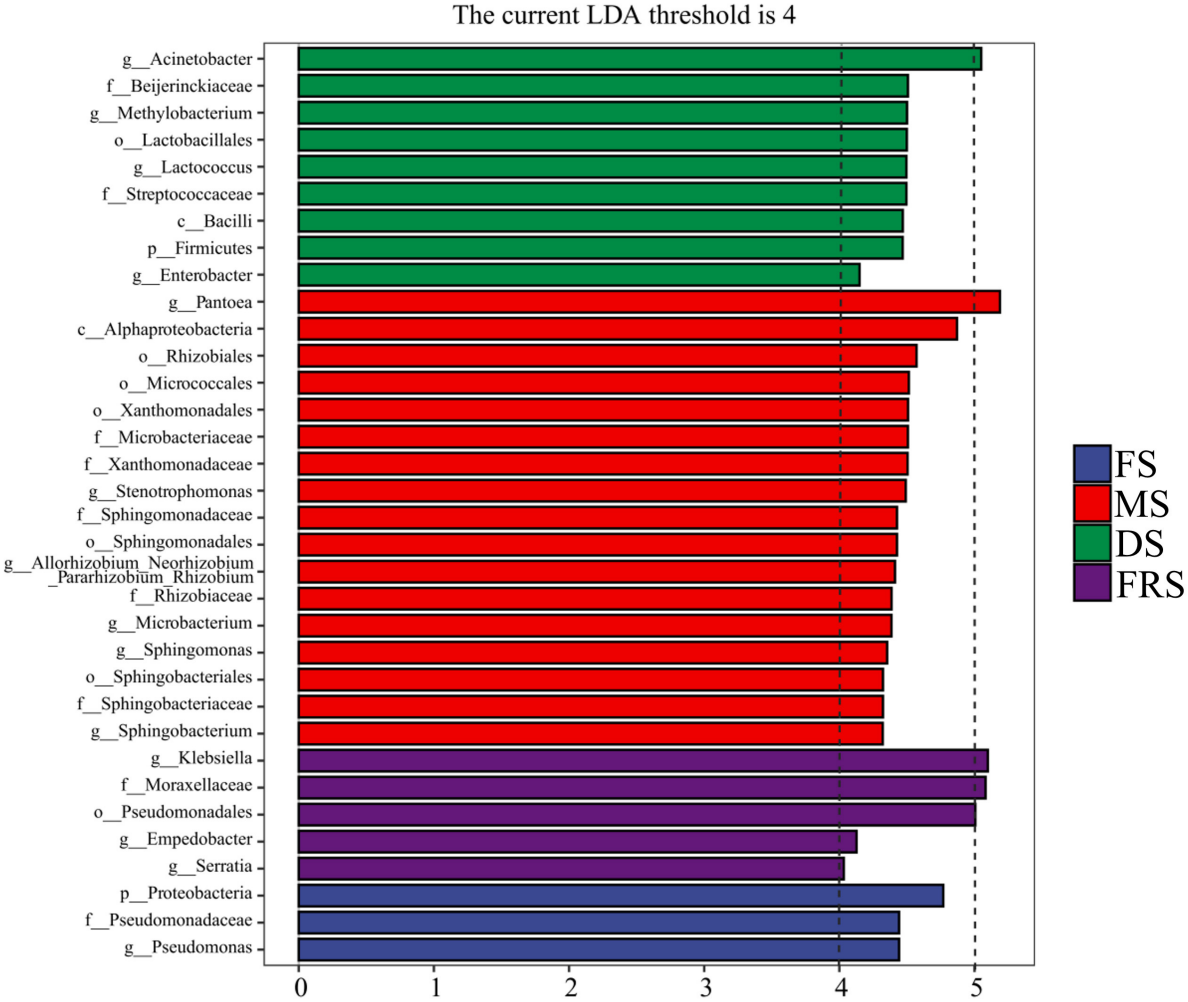
LEfSe multilevel species difference discriminant analysis of epiphytic bacteria at different growth stages. p, phylum level; c, class level; o, order level; f, family level; g, genus level. FS, flowering stage; MS, milk ripening stage; DS, dough stage; FRS, full ripening stage.

The inter-group LEfSe from phylum to genus level was performed on the basis of an Linear Discriminant Analysis (LDA) score >4, as shown in Figure 5. Proteobacteria had a greater influence on the difference between groups at the phylum level, and its relative abundance was higher in FS. The genus with the greater influence was *Pantoea*. *Pseudomonas* had a higher relative abundance in FS. *Pantoea, Stenotrophomonas, Allorhizobium–Neorhizobium–Pararhizobium–Rhizobium, Microbacterium, Sphingomonas*, and *Sphingobacterium* had a high relative abundance in MS. *Acinetobacter, Methylobacterium, Lactococcus*, and *Enterobacter* had a high relative abundance in DS. *Klebsiella, Empedobacter*, and *Serratia* had a high relative abundance in FRS.

## 4 Discussion

The dynamic change in the quantity of epiphytic microorganisms in the whole growth stage of rice was analyzed by using the coating plate method. The results showed that there were differences in the quantity of LAB, yeast, mold, and aerobic bacteria in different growth stages, and the quantity increased with the advancement of the growth stage, among which yeast was more at the milk ripening stage, mold was more at the full ripening stage, and LAB and aerobic bacteria were more at the flowering stage. Yin et al. (2023) found that the quantity of epiphytic microorganisms of Italian ryegrass (*Lolium multiflorum*) increased with the progress of the growth stage, and the quantity of LAB and enterobacteria was 4.33 and 5.34 lg10 CFU/g FM at the filling stage, and there were 5.42 and 6.01 lg10 CFU/g FM at the dough stage. Lin et al. (1992a) found that the epiphytic LAB on the alfalfa surface increased with the progress of the growth stage, and the quantity was higher in summer than in other seasons. This may be related to factors such as plant surface nutrients (Chen et al., 2023). The results showed that the water-soluble carbohydrate content of plants increased gradually with the progress of the growth stage (Wang T. et al., 2022), and the LAB was positively correlated with the soluble sugar content (Tang et al., 2021). However, the quantity of epiphytic LAB and aerobic bacteria gradually decreased in the mature stage (milk ripening stage, dough stage, and full ripening stage), which may be due to the dry matter content of the plant gradually increased from flowering to full ripening stage, and too high dry matter would inhibit the growth of LAB. In addition, the mold and yeast increase with the progress of the growth stage, and they will compete with them for nutrients, which may lead to the decrease of the LAB and aerobic bacteria (Zhao M. et al., 2021).

It was also found that since the flowering stage, the quantity of epiphytic microorganisms on the rice ear was more than that on the stem and leaf, which could be because a lot of photosynthetic products were transferred from the stem sheath to the ear after rice headed. Therefore, the contents of non-structural carbohydrates (mainly water-soluble carbohydrates and starch) in the stem sheath decreased sharply after the heading stage, which was lower than that of the rice ear (Liang et al., 1994). In this research, the quantity of aerobic bacteria in each growth stage was the highest, which was also consistent with the results of Cai et al. (1999) and Guan et al. (2018). Many aerobic bacteria and some harmful microorganisms such as yeast and mold on the surface of silage raw materials consume the residual oxygen and use the nutrients of raw materials to grow and reproduce in the early stage of silage, reducing the quality of silage (Xie et al., 2021). With the proliferation of LAB, the lactic acid produced can rapidly reduce the environmental pH value, inhibit the growth of undesirable microorganisms, and improve the silage quality. Kaiser and Weiss (1997) have found that the quantity of the epiphytic LAB of raw materials must reach 5 lg10 CFU/g FM to meet the requirement of improving the fermentation process of silage. In general, the quantity of LAB at the flowering stage, milk ripening stage, dough stage, and full ripening stage was more than 5 lg10 CFU/g FM and then had the potential to be used as whole rice silage. Therefore, in this research, high-throughput sequencing technology was used to sequence the rice epiphytic microorganisms at these four growth stages. The results showed that the composition and abundance of rice epiphytic microorganisms changed with the advancement of the growth stage.

At the phylum level, the composition of rice epiphytic microorganisms was Proteobacteria, Bacteroidetes, Actinobacteria, and Firmicutes. Proteobacteria dominated in different growth stages, with a relative abundance of >67%, which is basically consistent with the previous reports (Ali et al., 2020; Yuan et al., 2020). At the genus level, the top 10 dominant bacteria with relative abundance include *Pantoea, Acinetobacter, Chryseobacterium*, and *Methylobacterium*, as well as *Enterobacter, Klebsiella*, and *Serratia*, and most of them were undesirable microorganisms, which were supported by McGarvey et al. (2013). In addition, Knief et al. (2012) and Rastogi et al. (2012) found that a large quantity of *Methylobacterium* and *Pseudomonas* was found on the surface of rice and lettuce (*Lactuca sativa* var. *ramosa* Hort.). In the aerobic respiration stage of silage fermentation, microorganisms such as *Pseudomonas* and *Escherichia* attached to the surface of silage raw materials began to use the protein and carbohydrate for reproductive metabolism (Ávila and Carvalho, 2019) and break down carbohydrates into amino acids, acetic acid, and CO_2_ and water, which reduces the silage quality of feed (Borreani et al., 2018). Moreover, relevant studies showed that these harmful microorganisms can degrade proteins and nitrates and produce substances such as ammonia, biogenic amines, nitrite, and nitric oxide, which have an impact on silage quality and animal health (Schmithausen et al., 2022). This may explain the poor quality of rice silage in conventional silage; therefore, it is necessary to select superior epiphytic LAB to be added to rice silage to improve its fermentation quality and nutritional value.

Guan et al. (2021) found that there were fewer LAB on fresh corn, and only a small amount of *Lactococcus* was detected. Nazar et al. (2021) detected the epiphytic microorganisms of the whole corn plant and found that the dominant bacteria were *Enterobacter* (32.8%), and LAB were dominated by *Lactobacillus* (4.42%), *Weissella* (1.73%), *Leuconostoc* (1.29%), and *Lactobacillus* (0.02%). Yin et al. (2023) found that the dominant bacteria at the filling stage of Italian ryegrass were *Pseudomonas, Sphingomonas*, and *Microbacterium*. At the dough stage, the dominant bacteria were *Exiguobacterium, Allorhizobium, Pantoea*, and *Lactococcus*. In this research, only *Lactococcus* with high abundance was detected at the dough stage, but with lower abundance at the full ripening stage, and not detected at the flowering stage or the milk ripening stage. It is possible that epiphytic microorganisms of rice at the dough stage were beneficial to the fermentation process of silage.

In the composition of bacteria at different growth stages, *Pantoea* had a higher relative abundance at the milk ripening stage, while *Acinetobacte*r, *Chryseobacterium*, and *Methylobacterium* had a higher relative abundance at the dough stage. *Allorhizobium–Neorhizobium–Pararhizobium–Rhizobium* and *Stenotrophomonas* existed at the flowering stage and the milk ripening stage. However, *Microbacterium* exists only at the milk ripening stage. *Lactococcus* and *Enterobacter* were detected at the dough stage and the full ripening stage. *Klebsiella* was not detected at the milk ripening stage; *Serratia* was detected only at the full ripening stage. According to the above results, the epiphytic microorganisms of rice at different growth stages were dominated by Proteobacteria, Bacteroidetes, Actinobacteria, and Firmicutes at the phylum level, among which Proteobacteria are absolutely dominant, but there is a large difference at the genus level, which may be caused by climate and maturity (Dong et al., 2022; Zhao J. et al., 2021).

In addition to the growth stage, the growing conditions of plants also affect the diversity of epiphytic microorganisms. Guan et al. (2018) found that rainfall and temperature could affect the epiphytic microorganisms in corn raw materials. There was a significant positive correlation between precipitation and the relative abundance of *Lactobacillus, Acetobacter, Lactococcus*, and *Leuconostoc* in silage raw materials. Temperature was negatively correlated with the relative abundance of *Methylobacterium, Sphingomonas, Aureimona*s, and *Devosia*. In addition, there were certain differences in the diversity of epiphytic microorganisms in different growing regions. For example, Yuan et al. (2020) found that *Enterococcus* and *Pantoea* were the dominant bacteria on the surface of fresh alfalfa in Nanjing, China. Zhang et al. (2022) found that *Planococcus, Pantoea, Kocuria, Staphylococcus*, and *Corynebacterium* were the dominant bacteria on the surface of fresh alfalfa in Ningxia, China. McGarvey et al. (2013) found that *Erwinia, Escherichia*, and *Pseudomonas* were dominant bacteria on the surface of fresh alfalfa in California, USA.

## 5 Conclusion

There were great differences in the quantity and composition of epiphytic microorganisms in different growth stages of rice. The quantity of epiphytic microorganisms gradually increased with the progress of the growth stage, among which there were more aerobic bacteria on the surface of rice, while there were less LAB before the flowering stage. From the beginning of flowering stage, the quantity of LAB was more than 5 lg CFU/g FM. In terms of composition, the epiphytic microorganisms in different growth stages were mainly Proteobacteria, Bacteroidetes, Actinobacteria, and Firmicutes at the phylum level, among which Proteobacteria were absolutely dominant. At the genus level, mostly, enterobacteria such as *Pantoea, Enterobacter*, and *Klebsiella* and other harmful bacteria such as *Acinetobacter* and *Chryseobacterium* are found on rice surface. Only a high abundance of *Lactococcus* appeared at the dough stage, which may be beneficial to the silage fermentation utilization of rice at the dough stage. However, there are few relevant studies on the effects of epiphytic microorganisms at different growth stages on the fermentation quality of rice silage, combined with other existing studies, such as soybean (Bachmann et al., 2022), Italian ryegrass (Yin et al., 2023), millet (Zhao et al., 2024), and alfalfa (Wang S. et al., 2022). It can be seen that there are certain differences in the quantity and composition of epiphytic microorganisms at different growth stages, and they determine the fermentation quality of silage raw materials.

## Data Availability

The data presented in this study is stored in the NCBI SRA database, accession number PRJNA1177356.
