# Supplementary material for: Dynamic succession of the quantity and composition of epiphytic microorganisms at different growth stages on rice surface
Source: Front Microbiol. 2024 Nov 7;15:1451935. doi: 10.3389/fmicb.2024.1451935 (PMC11578753; doi:10.3389/fmicb.2024.1451935)
Supplement: Supplementary file 1 [file Table_1.docx]

| Original sample name | Latest sample name | Original sample name | Latest sample name |
| --- | --- | --- | --- |
| YHA1 | FS1 | RSA1 | MS1 |
| YHA2 | FS2 | RSA2 | MS2 |
| YHA3 | FS3 | RSA3 | MS3 |
| YHB1 | FS4 | RSB1 | MS4 |
| YHB2 | FS5 | RSB2 | MS5 |
| YHB3 | FS6 | RSB3 | MS6 |
| YHC1 | FS7 | RSC1 | MS7 |
| YHC2 | FS8 | RSC2 | MS8 |
| YHC3 | FS9 | RSC3 | MS9 |
| YHD1 | FS10 | RSD1 | MS10 |
| YHD2 | FS11 | RSD2 | MS11 |
| YHD3 | FS12 | RSD3 | MS12 |
| YHE1 | FS13 | RSE1 | MS13 |
| YHE2 | FS14 | RSE2 | MS14 |
| YHE3 | FS15 | RSE3 | MS15 |
| LSA1 | DS1 | WSA1 | FRS1 |
| LSA2 | DS2 | WSA2 | FRS2 |
| LSA3 | DS3 | WSA3 | FRS3 |
| LSB1 | DS4 | WSB1 | FRS4 |
| LSB2 | DS5 | WSB2 | FRS5 |
| LSB3 | DS6 | WSB3 | FRS6 |
| LSC1 | DS7 | WSC1 | FRS7 |
| LSC2 | DS8 | WSC2 | FRS8 |
| LSC3 | DS9 | WSC3 | FRS9 |
| LSD1 | DS10 | WSD1 | FRS10 |
| LSD2 | DS11 | WSD2 | FRS11 |
| LSD3 | DS12 | WSD3 | FRS12 |
| LSE1 | DS13 | WSE1 | FRS13 |
| LSE2 | DS14 | WSE2 | FRS14 |
| LSE3 | DS15 | WSE3 | FRS15 |

Annexed table 1 The correspondence between original sample names and sample names in the manuscript
